# A review on effect and treatment of perinatal *Ureaplasma urealyticum* colonization/infection in neonates

**DOI:** 10.1097/MD.0000000000041561

**Published:** 2025-02-21

**Authors:** Mengmeng Zhang, Yingjie Wang, Yuqian Wang, Junli Liu, Yanhong Wang, Li Zhang

**Affiliations:** a The Second Affiliated Hospital of Dalian Medical University, Shahekou District, Dalian City, Liaoning Province, China; b The Fifth People’s Hospital of Dalian, Shahekou District, Dalian City, Liaoning Province, China.

**Keywords:** neonatal diseases, preterm birth, treatment, *Ureaplasma urealyticum*

## Abstract

*Ureaplasma urealyticum* (UU) is the most frequently detected in the maternal genitalia, is mainly transmitted through vertical transmission at birth to neonates, with its complex etiology and adverse effects on neonatal health. While its etiologic significance in many aspects of neonatal diseases remains unknown, recent evidence indicates that UU is a cause of low birth weight, bronchopulmonary dysplasia, neonatal meningitis, retinopathy of prematurity, necrotizing enterocolitis, and abnormal blood cell counts, and is closely related to long-term prognosis in newborns. However, the most critical need is additional information concerning indications for treatment as well as the efficacy. This article reviews recent research progress in the effect and treatment of perinatal UU colonization/infection in neonates in order to provide evidence for the treatment of UU colonization/infection in neonates.

**Key Points**

- UU is transmitted through vertical transmission to neonates.
- UU infection can lead to adverse neonatal outcomes.
- Effective treatment for UU infection is necessary.

## 1. Introduction

*Ureaplasma* urealyticum (UU), a common commensal pathogen associated with maternity genital tract infections, is mainly transmitted through vertical transmission at birth to neonates. A growing number of studies have indicated that maternity UU genital colonization/infection can result in perinatal complications (abortion, premature delivery, and stillbirth) but also lead to adverse neonatal outcomes such as low birth weight, bronchopulmonary dysplasia (BPD), neonatal meningitis, and retinopathy of prematurity (ROP).

In this review, we summarize recent research progress in the effect and treatment of perinatal UU colonization/infection in neonates. Our hope is that these findings will provided evidence for the treatment of UU colonization/infection in neonates.

## 2. Characteristics and pathogenesis of UU infection

UU, the smallest known microorganism in humans, belongs to the family Mycoplasmataceae, class Mollicutes, order Mycoplasmatales and is commonly isolated from the urogenital tract. UU is divided into *U urealyticum* and *U parvum* comprising of 14 serovars. *U urealyticum* (genome is 16s rRNA) includes serovars 2, 4, 5, and 7 to 13, whereas *U parvum* (genome is 16-23s rRNA) includes serovars 1, 3, 6, and 14.^[1]^ The UU genome codes for multiple banded antigen IgA protease and urease. UU has no cell wall; thus, it is resistant to antibiotics that target cell-wall synthesis. It can also metabolize urea for ATP production.

**Figure 1.**
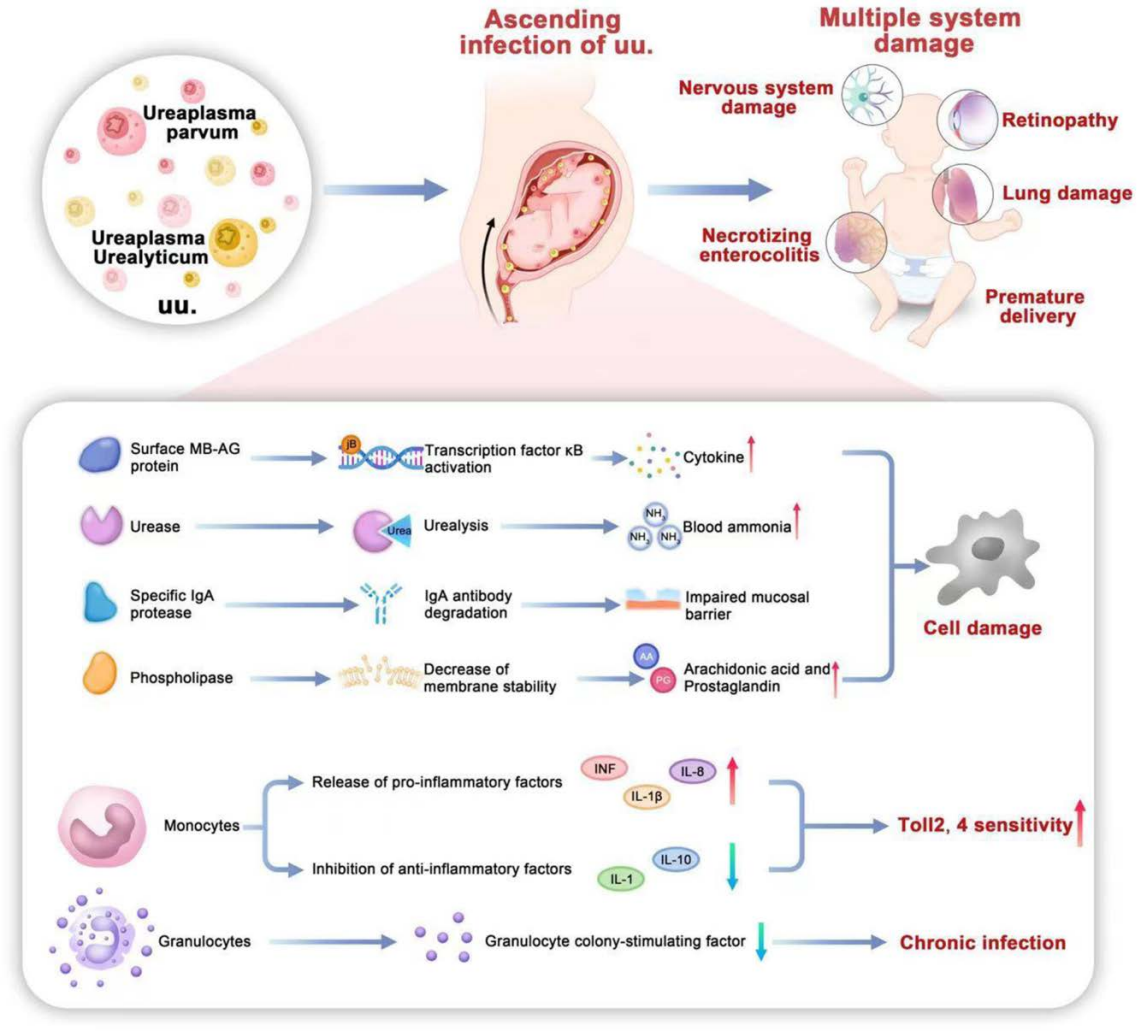
The pathogenesis of UU infection (UU includes *U urealyticum* and *U parvum* and is a commensal of the urogenital tract. Amniotic cavity colonization with UU can induce chorioamnionitis. Neonates may be infected with UU by vertical transmission. The pathogenic mechanism of UU mainly include: (1) MBA protein can be recognized by host cells through TLR, activate transcription factor κB to increase cytokine. (2) UU contains urease, and urease metabolism results in hyperammonemia. (3) Specific IgA proteases can degrade IgA antibodies leading to mucosal barrier damage. (4) Phospholipases can degrade host cell membranes producing arachidonic acid and subsequently prostaglandins. (5) UU infection can cause cytokine imbalance resulting in chronic infection by inducing a decrease in colony-stimulating factors.). MBA = multiple banded antigen, TLRs = Toll-like receptors, UU = *Ureaplasma urealyticum*.

Currently, the pathogenesis of UU infection is as follows: first, UU may escape the host immune response via phase and size variations in the multiple banded antigen protein (which is unique to UU). Because of its similarity with the human salivary duct epithelium, vas deferens epithelium, IgA Fc receptor, and DNA-binding protein, UU can be recognized by the host, absorbing lipids and cholesterol and increasing cytokine levels by activating transcription factor κB via Toll-like receptors (TLRs), resulting in the development of UU infection.^[2]^ Second, urease metabolism results in hyperammonemia, which can lead to tissue damage.^[3–5]^ Lastly, specific IgA proteases can degrade IgA antibodies (leading to mucosal barrier damage) and may also lead to the escape of UU from the host’s immune system. However, further studies are required to identify the genes encoding IgA protein.^[2]^ Phospholipases contribute to this pathogenesis by degrading host cell membranes via phospholipid metabolism and the production of arachidonic acid (and subsequently prostaglandins).^[6]^ UU has been identified as contributes to immune dysfunction by causing imbalances in cytokine levels. Several studies have shown that the host produces a significant inflammatory response after exposure to UU, as monocytes increase the secretion of pro-inflammatory factors (TNF-α, IL-1β, IL-6, and IL-8) and decrease the secretion of anti-inflammatory factors (IL-1 receptor antagonists and IL-10). In addition, increased susceptibility of TLRs-2 and TLRs-4 to pro-inflammatory factors has been observed.^[7,8]^ In addition, UU infection may decrease the serum levels of granulocyte colony-stimulating factors, thereby reducing immune defense and resulting in the long-term survival of UU and chronic infection.^[9]^ The pathogenesis of the UU infection is shown in Figure 1.

## 3. The vertical transmission rate of UU and colonization rate in newborns

UU, which has an average colonization rate in maternal genitalia of 40% to 80%, has been confirmed to be associated with chorioamnionitis (which can lead to fetal intrauterine infection). Neonates can be infected via vertical transmission,^[10–12]^ and the pathogen can colonize the respiratory tract, digestive tract, central nervous system, and bloodstream.^[13]^ It was also reported that the lower the gestational age, the higher the vertical transmission rate, and the vertical transmission rate approaches 90% in neonates weighing <1 kg, it is not affected by the delivery mode of the neonate or the time of premature rupture of membranes.^[1]^ A study on UU colonization demonstrated that 35% of pregnant women had UU colonization in genitalia and 55% of infants had at least 1 culture site positive for UU within 3 days after birth (throat, 41%; eyes, 20%; vagina, 40%).^[14]^ Another study demonstrated that the UU positivity rate in nasopharyngeal secretions of neonates was 11%, the UU colonization rate in the vaginas of pregnant women was 15%, and the vertical transmission rate was 72%.^[15]^ A study on the vertical transmission of UU in full-term neonates without respiratory symptoms found that the UU positivity rate in pregnant women was 42%, including 36% for *U parvum* and 6% for *U urealyticum*. The UU positivity rate in the tracheal suction fluid of neonates was 17.4%, while the vertical transmission rate was 38%. The relative vertical transmission rates of *U parvum* and *U urealyticum* were 33 and 67%, respectively.^[16]^

## 4. Laboratory testing of UU

The most commonly applied detection methods are culture and polymerase chain reaction (PCR) assays. However, the use of metagenomic next-generation sequencing is increasing.

### 4.1. Culture

Culture is the gold standard or detecting UU, and can provide date of drug-sensitives. However, culture takes a long time, and the UU positivity rate is low because it requires serum, metabolic substrates, and growth factors for isolation.^[17]^

### 4.2. PCR assays

The modified PCR assays have the ability of rapid detection of UU infection. They are more sensitive, specific than the culture method. The sensitivity and PPV of the real-time fluorescence LAMP assay has been reported to be 100% (16/16 specimens; 95% CI 79.4–100%) and 100% (16/16; 95% CI 79.4–100%) compared to culture results.^[18]^ PCR assays are used to analyze large samples and are especially appropriate for organisms at low concentrations.

### 4.3. Metagenomics next-generation sequencing

Metagenomic next-generation sequencing, a laboratory diagnostic technology based on high-throughput sequencing, can sequence all the biological genomes of various clinical samples. It has the advantages of unbiasedness, wide coverage, high sensitivity, and relatively rapid pathogen identification. Thus, it is used in the diagnosis of various infectious diseases.^[19]^ And in Okumura et al study, UU has been detected in the gastric fluid of neonates with respiratory distress and chorioamnionitis by next-generation sequencing.^[20]^

## 5. The effect of UU on neonatal diseases

The mechanism of neonatal UU contamination involves intrauterine infection or intrapartum transmission. Neonatal UU infection is usually followed by adverse neonatal outcomes, such as low birth weight, neonatal pneumonia, BPD, and meningitis.^[21]^ A prospective observational multicenter study of 4330 pregnant women with a gestational age ≤ 33 weeks demonstrated that vaginal UU colonization was significantly associated with severe intraventricular hemorrhage (IVH) (10.4% vs 2.6%), ROP (21.7% vs 10.3%), and adverse psychomotor outcomes (24.3% vs 1.8%),^[12]^ suggesting a potential role for UU in neonatal diseases. A better understanding of UU infection and its role will offer insights into the early diagnosis and effective treatment of infection to prevent long-term sequelae.

### 5.1. Preterm birth and low birth weight

Approximately 15 million preterm infants are born annually worldwide, with a global preterm birth rate of approximately 11%. Despite improvements in healthcare, the incidence of preterm births has not declined.^[22]^ In approximately one-third of the cases, preterm birth is attributed to infection in the amniotic cavity.^[23]^ UU is most frequently isolated from the amniotic cavity following preterm labor and delivery, suggesting an association with preterm.^[10,24]^ Judith et al reported that in approximately 10.4% of cases, vaginal colonization in pregnant women with *U parvum* occurred alongside spontaneous preterm birth (SPB), whereas in 6.4% of cases, preterm birth occurred in women with no UU vaginal colonization. They also reported that the lower the gestational age, the higher the *U parvum* colonization rate, suggesting that vaginal colonization by *U parvum* in early pregnancy is an independent risk factor for SPB.^[10]^ A study of vaginal *U parvum* serovars and SPB demonstrated that 25.2% of the samples were positive for serovar 1, 43.3% of the samples were positive for serovar 3, and 31.4% of the samples were positive for serovar 6. In addition, SPB occurred in 10.5%, 10.9%, and 10.5% of pregnancies with serovar 1, 3, and 6 colonization, respectively, which was significantly higher than the negative results. Serovar 3 had the most significant association with vaginal *U parvum* colonization and preterm delivery, and the lower the gestational age, the greater the difference in SPB.^[13]^ Kwak et al showed that pregnant women carrying both types of UU biota had significantly lower gestational age and birth weight.^[21]^ Furthermore, women infected with UU have a higher risk of cesarean section due to preterm delivery or premature rupture of membranes, and their fetuses were more likely to have low birth weight.^[25,26]^

UU colonization may result in preterm birth through the activation of host immune defense mechanisms, resulting in expulsion of infected tissues to protect reproductive health. UU is also associated with the production of pro-inflammatory factors (IL-6, IL-8, and TNF-α), resulting in systemic inflammatory responses. In addition, the synthesis and release of prostaglandins can stimulate uterine contractions, leading to the initiation of preterm labor.^[22,27,28]^ Although most previous studies have found that UU is associated with adverse pregnancy outcomes, the pathogenesis of this finding is unclear. Thus, further studies are required to determine the pathogenesis of UU-induced preterm birth.

### 5.2. Neonatal respiratory diseases

#### 5.2.1. Pulmonary inflammation in neonates

Investigations in which UU was isolated from the respiratory tract of newborns have suggested that it plays an important role in the pathogenesis of neonatal respiratory diseases. A study by Maria Agnese Latino demonstrated that the detection of UU in placental samples was significantly associated with acute chorioamnionitis compared to UU-negative samples, and the proportion of fetal pneumonia was significantly higher among samples positive for acute chorioamnionitis than among negative controls.^[29]^ Kotecha et al reported significantly increased counts and IL-6 titers in bronchoalveolar lavage fluid samples from UU-positive preterm infants (24 weeks ≤ gestational age ≤ 29 weeks) in the early stage and gradually increased macrophage counts and intercellular adhesion molecules with the development of pneumonia.^[30]^ Increased levels of inflammatory factors in bronchoalveolar lavage fluid after UU infection have also been confirmed in animal models. Jamile et al demonstrated that neutrophil counts and pro-inflammatory cytokine (IL-1β and TNFα) levels in bronchoalveolar lavage fluid samples significantly increased 72 hours after UU infection and gradually decreased at 7 and 14 days post-infection.^[31]^ These studies suggested that UU plays an important role in the pathogenesis of acute pulmonary inflammation in neonates.

#### 5.2.2. Neonatal respiratory distress syndrome (NRDS)

Several studies have reported a significantly higher prevalence of UU infection in neonates with NRDS than in those without it.^[32]^ A previous study demonstrated that nuclear factor κB activation in lung neutrophils and macrophages in UU-positive neonates and infants with activated nuclear factor κB resulted in elevated TNF-α concentrations in their tracheal aspirates. As a transcription factor that regulates the inflammatory process, NF-κB activation in pulmonary leukocytes may be involved in the pulmonary inflammatory process in neonatal acute respiratory distress syndrome.^[32]^ Contrary to clinical research, animal studies have shown that *U parvum* infection increases the expression of surfactant protein A and surfactant protein B, and increases lung volume.^[33]^ It has been suggested that UU infection increases pulmonary surfactant levels and may contribute to fetal lung maturation; thus, UU infection could be a protective factor against NRDS. The contradictions between clinical and animal studies may be related to the following aspects. First, NRDS is affected by multiple factors including gestational age, lung maturation, and infection. Therefore, it is difficult to exclude other factors that may influence the occurrence and pathogenesis of NRDS. Second, in clinical practice, the diagnosis of NRDS is largely influenced by doctors’ subjective impressions, which hinders objectivity. Moreover, the small sample size is a limitation that may have led to biased results. Further studies are required to explore the correlation between UU infections and NRDS.

#### 5.2.3. BPD

With the development of medical treatment, the survival rate of extremely preterm infants has increased; thus, the incidence of BPD, a sequela of preterm infants, is also increasing. Recent studies have focused on the association between UU and BPD. Several studies have confirmed that UU colonization is an independent risk factor for BPD in very low birth weight infants.^[34,35]^ Glaser et al reported a significantly higher prevalence of BPD in UU-positive infants whose duration of ventilator use exceeded 5 days than in UU-negative infants.^[34]^ UU infection may reduce the levels of vascular endothelial growth factor, angiopoietin 1, and vascular endothelial growth factor receptor 2, leading to a reduction in vascular density and abnormal pulmonary vascular development, resulting in pulmonary dysfunction and development of BPD.^[33,36]^ In addition, UU infection may upregulate the expression of matrix metalloproteinase-9, which may increase the levels of pro-inflammatory factors (monocyte chemoattractant protein 1, monocyte chemoattractant protein 3, etc), leading to airway remodeling and abnormal alveolar development, followed by the development of BPD.^[37]^

However, contrary to the findings of the above studies, a previous study showed that comparisons of the duration of oxygen and ventilator use between infants with SPB caused by UU infection, infants with SPB caused by other factors, and electively born preterm infants suggested that UU infection was not an independent risk factor for BPD.^[38]^ These contradictory conclusions may be because the pathogenesis of BPD is related to multiple factors, such as infection, mechanical ventilation, and gestational age. In addition, the small number of samples, inconsistent diagnostic criteria for BPD, different sampling tissues, and different grouping methods also led to contradictory conclusions. Further studies are needed to clarify the relationship between UU infections and BPD.

### 5.3. Effects on blood cells

Several clinical studies have reported that UU infection can lead to abnormal blood cell test results in newborns. Ohlsson et al reported that leukocyte and neutrophil counts in the lower respiratory tract were significantly increased in the peripheral blood of preterm infants infected with UU in the lower respiratory tract.^[39]^ A study of preterm infants (gestational age ≤ 32 weeks and birth weight ≤ 2000 g) demonstrated that leukocyte and platelet counts in UU-infected infants were significantly higher than those in uninfected infants. This study suggested that postnatal leukocyte count was an independent risk factor for UU infection, and leukocyte counts in postnatal hemograms of premature infants could be used as a diagnostic marker for UU infection.^[40]^ Therefore, attention should be paid to the possibility of UU infection in newborns with unexplained increases in leukocyte and platelet counts. However, there is a paucity of reports on the blood cell count abnormalities caused by UU. Leukocytosis, leukemia-like reactions, anemia, and platelet abnormalities caused by UU infections are common in clinical practice. Therefore, further studies are required to explore the effects of UU infection on neonatal blood cells.

### 5.4. Neonatal nervous system diseases

Currently, there are many reports of neonatal nervous system diseases caused by UU infections. It has been suggested that UU may be the causative pathogen of neonatal central nervous system infections. The lower the gestational age, the greater the possibility of central nervous system injury. Jepp et al reported reported a case of UU meningitis complicated by hydrocephalus in a preterm neonate born at 26 + 3 weeks of gestation. The infant developed frequent seizure activity that were resistant to anti-seizure medications. UU was detected in the cerebrospinal fluid (CSF) and treatment was performed using a combination of azithromycin and ciprofloxacin. When the pathogen is cleared from the CSF, seizure acitivity generally stopped.^[41]^ Viscardi et al reported a 19.1% UU contamination rate in the CSF of very low birth weight infants born at ≤ 33 weeks of gestation. This study demonstrated that the risk of severe IVH (grade > 3) was 2.32 times higher in PCR-positive infants than in PCR-negative infants, and the detection rate of *U parvum* was higher than that of *U urealyticum* in IVH infants.^[42]^ Olomu et al reported a significantly increased risk of IVH and echolucent brain lesions in preterm infants (gestational age ≤ 28 weeks) when UU was detected in their placental parenchyma of their mothers.^[43]^ In addition, a clinical study found that the isolation of UU from the amniotic cavity at birth was significantly associated with abnormal psychomotor development and adverse neuromotor outcomes, increasing the risk of cerebral palsy at 1 and 2 years of adjusted age in preterm infants.^[44]^

The neuroinflammatory potential of UU may be related to the following factors. First, UU infection may activate the systemic inflammatory response, stimulating the production of inflammatory factors and provoking abnormal neuronal development, with delayed myelination and microglial activation, followed by abnormal development of the nervous system. In addition, UU exposure may decrease granulocyte colony-stimulating factor levels and increase vascular endothelial growth factor levels, increasing cerebrovascular brittleness and blood–brain barrier permeability, which, in turn, facilitates the entry of pathogens into the central nervous system and facilitates inflammatory cell influx, ultimately promoting neuroinflammation.^[45–48]^

Several previous studies have reported that UU infection is closely related to neonatal nervous system injury. It is important to pay attention to the screening and treatment of UU to reduce neonatal nervous system injuries and to prevent long-term sequelae.

### 5.5. Neonatal necrotizing enterocolitis (NEC)

NEC, a disease that causes intestinal mucosal damage for multiple reasons, is characterized by diffuse necrosis of the small intestine and the colon. This condition is known as a gastrointestinal emergency. Several studies have reported that UU is detected in the gastric aspirates and rectal samples of preterm infants and may play a role in the pathogenesis of NEC. Okogbule-Wonodi et al reported that the incidence of NEC was 2.2 times higher in UU-positive infants (12.3%) than in UU-negative infants born at ≤ 33 weeks of gestation (5.5%) and 3.3 times higher in UU-positive infants (14.6%) than in UU-negative infants (4.4%) born at ≤ 28 weeks of gestation.^[49]^

Recent studies have speculated that UU could contribute to the pathogenesis of NEC by facilitating the reduction of the concentrations of specific intestinal proteins and related products, which causes the loss of intestinal neurons and glial cells, leading to enteric nervous system dysfunction.^[50]^ In addition, immature intestinal function in preterm infants may increase their susceptibility to UU, leading to intestinal inflammation with damaged epithelial villi, intestinal barrier loss, and severe villus atrophy, ultimately causing gastrointestinal dysfunction.

### 5.6. ROP

ROP is a retinal vascular disease in preterm infants and a major cause of childhood blindness.^[51]^ ROP is a multifactorial disorder, with low gestational age, low birth weight, and oxygen exposure being major risk factors.^[52,53]^ Growing evidence suggests that UU infection plays a role in ROP pathogenesis. A study on ROP infants with birth weights ≤ 1250 g showed that 52% of those with severe ROP were culture-positive for UU, whereas only 30.4% of those with either mild or no ROP were culture-positive. This suggests that BW, duration of mechanical ventilation, premature rupture of membranes > 18 h, and UU positivity are independent risk factors for the development of severe ROP.^[54]^ A Chinese study on intrauterine UU infection demonstrated that the incidence of ROP among UU-positive infants was higher than that among UU-negative infants.^[40]^ It is speculated that UU infection decreases birth weight and increases the demand for supplemental oxygen, thereby contributing to the development of ROP.

In conclusion, neonatal UU infection can lead to multisystem diseases. The pathogenicity of UU is determined by multiple factors such as biota, serovars, concentrations, and host immunity. Clinicians should pay attention to UU infections and perform timely diagnosis and treatment to avoid missing the best time for treatment (which would result in long-term sequelae).

## 6. Treatment of UU infection

UU does not have a cell wall and antibiotics targeting the cell wall (e.g., penicillin, cephalosporins, and vancomycin) are ineffective. UU is sensitive to antibiotics that can inhibit protein synthesis or topoisomerases, such as quinolones, tetracycline, and macrolides^[1]^; thus, macrolides are frequently used to treat neonatal UU infections. A randomized trial showed that the incidence of BPD is reduced by treatment with high dose of azithromycin.^[55]^ There has a case report suggesting that azithromycin may be effective in treating severe cerebral impairment caused by UU in a preterm neonate.^[41]^ However, mild cerebral impairment and other pulmonary morbidities did not decrease despite macrolide antibiotics.^[56]^ In addition, the safety of macrolide antibiotics requires further study, and there have been case reports on proarrhythmia, such as QT interval prolongation and *torsades de pointes*.^[57]^ Symptomatic support and management of complications are the main treatments for neonatal UU infections.

Nucleotide exchange factor, a highly conserved antigen, is rapidly expressed and upregulated when UU infects a host. A study conducted using nucleotide exchange factor-immunized mice demonstrated that nucleotide exchange factors can induce a strong immune response, which can reduce the UU burden in tissues without increasing inflammation in response to infection.^[58]^ This study suggests that the nucleotide exchange factor protein might be a promising new vaccine candidate for the prevention of UU infection.

## 7. Conclusion

In conclusion, UU colonization, a common problem during the perinatal period, leads to fetal infection through vertical transmission and adversely affects newborn health. In clinical practice, care should be taken to prevent and treat perinatal infections with *U urealyticum* infections to reduce the incidence of adverse outcomes after neonatal infection.

## Acknowledgments

The authors sincerely thank the pediatric teachers of the Second Affiliated Hospital of Dalian Medical University for their guidance, assistance, and concerns. They always help us with dribs and drabs of life, cheer me up, and provide encouragement when we lack confidence. The authors are also grateful to their families for their support and encouragement. Without their strength and confidence in my ability to study hard, the authors believe that this paper will be difficult to complete. Finally, the authors would like to thank all their classmates, friends, teachers, and relatives who have always cared for, supported, and helped them.

## Author contributions

**Formal analysis:** Yuqian Wang, Yanhong Wang.

**Supervision:** Yingjie Wang.

**Software:** Junli Liu.

**Writing – original draft:** Mengmeng Zhang, Yingjie Wang.

**Writing – review & editing:** Li Zhang.
